# Exploring Natura 2000 habitats by satellite image segmentation combined with phytosociological data: a case study from the Čierny Balog area (Central Slovakia)

**DOI:** 10.1038/s41598-022-23066-3

**Published:** 2022-11-01

**Authors:** Lucia Čahojová, Martin Ambroz, Ivan Jarolímek, Michal Kollár, Karol Mikula, Jozef Šibík, Mária Šibíková

**Affiliations:** 1 Institute of Botany, Plant Science and Biodiversity Center, Slovak Academy of Sciences, Dúbravská Cesta 9, 845 23 Bratislava, Slovakia; 2 Department of Mathematics, Slovak University of Technology, Radlinského 11, 810 05 Bratislava, Slovakia

**Keywords:** Biodiversity, Forest ecology

## Abstract

Natura 2000 is a network of protected areas covering Europe's most valuable and threatened species and habitats. Recently, biota belonging to these networks have been threatened by both climate change and various human impacts. Regular monitoring is needed to ensure effective protection and proper management measures in these sites and habitats, but conventional field approaches are often time-consuming and inaccurate. New approaches and studies with different focuses and results are being developed. Our approach includes point data from field research and phytosociological databases as starting points for automatic segmentation, which has been developed just recently as a novel method that could help to connect ground-based and remote sensing data. Our case study is located in Central Slovakia, in the mountains around the village of Čierny Balog. The main aim of our case study is to apply advanced remote sensing techniques to map the area and condition of vegetation units. We focus on forest habitats belonging mainly to the Natura 2000 network. We concentrated on the verification of the possibilities of differentiation of various habitats using only multispectral Sentinel-2 satellite data. Our software NaturaSat created by our team was used to reach our objectives. After collecting data in the field using phytosociological approach and segmenting the explored areas in the program NaturaSat, spectral characteristics were calculated within identified habitats using software tools, which were subsequently processed and tested statistically. We obtained significant differences between forest habitat types. Also, segmentation accuracy was tested by comparing closed planar curves of ground based filed data and software results. This provided promising results and validation of the methods used. The results of this study have the potential to be used in a wider area to map the occurrence and quality of Natura 2000 habitats.

## Introduction

Remote sensing (RS) is one of the most important tools in ecology and conservation for the effective monitoring of ecosystems in space and time^1^. Satellite remote sensing of ecosystem functions could offer many opportunities to advance environmental and nature protection, test emerging theories, and unveil the processes that shape the impacts of anthropogenic threats on biodiversity more rapidly^2^. Using satellite images for monitoring habitats and biota dynamics has been highlighted in many research activities. There is a large potential that is already at the beginning of this effort depending on the technical background (either hardware or software). Biodiversity conservation based on the conservation of seminatural and natural habitats has been one of the objectives of the European Biodiversity Strategy to 2020^3^. Since the Convention on Biological Diversity and the European Union Biodiversity Strategy to 2030 set ambitious targets for increasing the extent of protected areas^4^, developing reliable methods substituting expert knowledge and fieldwork is urgent. There are of course many challenges in such methodologies, including mapping ecotone transitions between vegetation types and/or low resolution of available satellite images. Remote sensing habitat monitoring can indicate core areas that are essential for key species and biodiversity patterns and transitional zones that are important for ecosystem processes^5^. Digital mapping techniques provide accurate maps that can also be used in climate models to assess the sensitivity and feedback to future climate change^6^. Mapping landscape-level heterogeneity of microclimate advances our ability to study how organisms respond to climate variation, which has important implications for understanding climate-change impacts on biodiversity and ecosystems^7^. The accurate and rapid extraction of vegetation cover information from an image enables the monitoring of vegetation changes over time, which is of great significance for protecting biodiversity, maintaining social stability, and promoting economic development^8^.

Land cover mapping requires a typology or classification by which the more-or-less continuous variations in element composition are sorted into discrete habitat or vegetation units^9^. Based on vegetation variability, differences in species composition that arise from a different environment and competition strategy between individual species can be further structured due to variation in vegetation phenology. It is a commonly used indicator signalling vegetation responses to global changes^10^. When comparing field expert-based approaches, there are individual inconsistencies despite using the same mapping systems, materials, and methods. The differences are usually more enormous at lower hierarchical levels in the mapping systems and increase strongly with system complexity^9^; therefore, a proper resolution is needed at the highest possible level depending on the intended goals. In contrast, many remote sensing images (aerial or satellite) of the target vegetation types can have certain disadvantages because of their potentially high cost, long cycles, and low resolution^8^.

From the previously mentioned examples, satellite remote sensing data could be used for mapping broader units, e.g., riparian vegetation in general^11^ or broadleaved deciduous forests versus coniferous forests^12,13^. The level of a single habitat or plant community on the alliance level could be reached by unmanned aerial vehicles (UAVs) or airborne laser scanning (ALS) photographs in the case of insufficient resolution of satellite images. The disadvantage of these high-resolution methods is their limitation to a small area, high cost, and usually one-time sampling without repetitions. Sentinel-2 satellite images cover large areas with a high revisit frequency^14^. The recognition of single habitats using Sentinel-2 data could give us a great opportunity to monitor Natura 2000 habitats on the European level and describe their spatiotemporal dynamics.

In Europe, there is an old tradition of collecting vegetation data that have recently been stored in the European Vegetation Archive database (EVA)^15^. These data contain point information about species composition and stand structure and can be directly transformed into habitat type^16^. The majority of European countries have their own national databases, e.g., the Slovak Vegetation Database^17^, that were created to collect vegetation data of their territories and to be as representative as possible. In lists of habitats and vegetation units^18–20^, maps of Natura 2000 habitat occurrence are presented as a square grid with information about habitat presence/absence derived from phytosociological relevés coordinates. However, these phytosociological relevés have a large potential to serve as a base for exact habitat mapping if they are transformed from point to spatial data using, e.g., remote sensing techniques.

Phenological aspects can play an important role in the recognition of land cover types and specific vegetation through remote sensing and can make their identification and classification more accurate. Several studies have been devoted to the added value of multiseasonal images, which capture different periods of the growing season, to characterize land cover types^21–26^. Dymond, Mladenoff^21^ used phenological information in satellite images and, as reported, together with vegetation indices, improved the classification of northern temperate forests. Phenology-based classification has also been used in several studies to map and monitor monocultures, e.g., rubber plantations^27–30^.

The satellite image segmentation methods used in the present study are one such tool^31–33^. They work based on evolving planar curves and are efficient and robust segmentation tools when an “initial estimate” of the desired area is available. In the case of Natura 2000 habitats, the pointwise estimate of habitat occurrence is available from vegetation databases or field surveys.

The aims of the present study are (i) to show the feasibility of recognizing Natura 2000 forest habitats based on optical information from Sentinel-2 satellite data; (ii) to find a suitable period of the year for habitat recognition based on phenological aspects; and (iii) to test the feasibility of using phytosociological relevés with coordinates as starting points for semiautomatic and automatic segmentation to find the exact habitat area.

## Methods

### Study area

**Figure 1:**
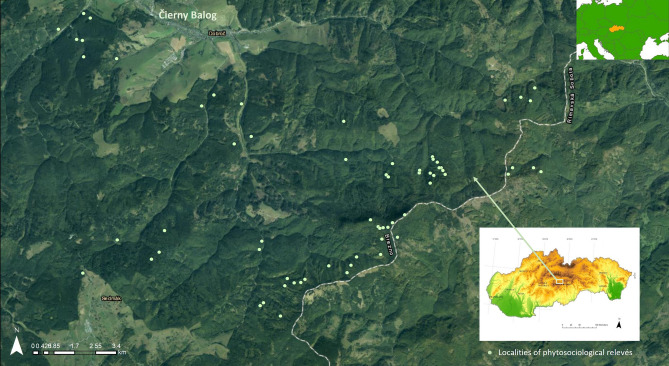
Localization of the case study area (created in ArcGIS ArcMap 10.4. Ortofotomozaika SR – ÚGKK ZBGIS 2021 https://zbgisws.skgeodesy.sk/zbgis_ortofoto_wmts/service.svc/get; data source ^37^).

The case study area is situated in the vicinity of the village of Čierny Balog in central Slovakia at an altitude of 559–1338 m a.s.l. (Fig. 1).

Čierny Balog is located in the Slovenské Rudohorie Mountains in the area of the Vepor Mountains and Balocké Vrchy Mountains formed by crystalline and Mesozoic rocks of the Veporidy geological unit^34^. The cadastral district of Čierny Balog corresponds with the water catchment area of Čierny Hron. Most of the area is moderately cold and very wet (temperature of 12 °C to 16 °C in July), and the surroundings of Klenovský Vepor (the highest part of the study area) are characterized by a cold mountainous and very humid climate (with a temperature of 10 °C to 12 °C)^35^.

The area was affected by two colonization phases (Wallachian since the fifteenth century and German since the sixteenth century), when parts of the ancient natural forests were cut down^36^. Later, wood processing and pastoralism were the main activities that contributed to the gradual transformation and deforestation of the study area.

According to the Habitats Directive^38^ and habitat classification of the Natura 2000 system in Slovakia (cf ^19^.), the study area is characterized mainly by forest habitats: 9130 *Asperulo-Fagetum* beech forests; 9180 *Tilio-Acerion* forests of slopes, screes, and ravines; and 9410 Acidophilous *Picea* forests of the montane to alpine levels (*Vaccinio-Piceetea*) (spruce forests) and planted spruce forest (secondary spruce forests).

### Data sampling

#### Phytosociological approach and GPS tracking

Together, 65 phytosociological relevés were sampled during field research in the summer of 2020 and 2021, proportionally covering individual habitat types in the study area. Each relevé was sampled on a 400 square metre plot using the Zürich–Montpellier School standard methodology^39–41^. The quantitative ratio of relevés of target habitats is represented by their presence in the region. Relevés were stored in the TURBOVEG database^42^ and processed in the JUICE programme^43^. The nomenclature of taxa follows Marhold et al.^44^. The nomenclature of syntaxa follows Jarolímek et al.^18^, and habitat names are under the Habitats Directive, formally known as Council Directive 92/43/EEC on the conservation of natural habitats and of wild fauna and flora^38^.

During the 2020 and 2021 vegetation seasons, the boundaries of 24 selected habitats (8 segments of 9130 *Asperulo-Fagetum* beech forests; 9180 *Tilio-Acerion* forests of slopes, screes, and ravines; and planted spruce forest), with a total area of 70.26 ha, were tracked by a GPS device, Garmin Oregon 600t. For this case study, forest segments with an easier definition of boundaries (which are surrounded by a different type of habitat) and areas with more complex boundaries (which border on the same or similar type of forest) were chosen. The compact stands of the native spruce forests (habitat 9410) were concentrated only in one locality, which did not allow us to sample an appropriate amount of data. Moreover, the occurrence of 9410 habitat in the study area was on its southern border of distribution, and habitat did not represent typical characteristics there. In the case of problems in the field (loss of GPS signal or overly steep impassable rocky slopes), GPS tracks were additionally corrected in Google Earth Pro software.

#### Segmentation methods

NaturaSat software integrates various image-processing techniques together with vegetation data management^31^. The software allows a user to focus the Sentinel-2 image to the selected habitat occurrence indicating point and then allows a user to perform either semiautomatic^32^ or automatic^33^ segmentation by evolving the initial curve, either in the form of a straight line or automatically chosen image isoline (semiautomatic segmentation) or in the form of a small circle (or circles) or a small square (automatic segmentation). The segmentation curve is evolved by a general mathematical model including homogeneity and edge detector driving forces and curvature influence^32,33,45^.

Semiautomatic segmentation requires user interaction. The user clicks the mouse at some correctly chosen point on the habitat boundary and drags the mouse along the expected habitat boundary—the algorithm always connects the first clicked point with the last mouse position, constructs the initial curve between them and adjusts this line to the habitat border in real time by using the numerical scheme^32,45^. The overall segmentation results are given by an interconnection of several open curve segments. The quality of these segments is controlled by the user and governed by the mathematical model and its parameters.

The automatic segmentation software tool is based on a similar principle but evolves closed planar curves in the Lagrangian formulation. A detailed description of the mathematical model, numerical implementation, and numerical experiments showing the behaviour of the developed method were presented by Mikula et al.^32,33^. The automatic segmentation could start from the user-defined initial closed curve or from the phytosociological relevé representing the current habitat or plant community.

In the present study, both types of segmentation methods were used to find a homogeneous area of approximately 65 phytosociological relevé that represents analysed habitats (with an area of 331.53 ha of polygons obtained semiautomatically and 306.53 ha obtained automatically). These 65 areas were supplemented by other segments based on field research and detailed knowledge of local forest vegetation and data from LGIS^46^. A total of 107 areas were segmented (with an area of 586.51 ha of polygons obtained semiautomatically), which were divided into four groups according to habitat type resulting from the species composition of phytosociological relevés and expert knowledge.

The automatic segmentation is influenced by the expanding term, including the homogeneity detector function. The values of these parameters could give different segmentation results in the case of different habitats. Usually, monodominant forests require a smaller min–max range of the homogeneity function in comparison with mixed forests. Tuning these parameters for the studied habitats was one of the aims of the present research.

#### Computing of the multispectral characteristics

NaturaSat tools allow us to compute spectral characteristics inside segmented regions. The characteristics include mean, minimal, and maximal intensity values and standard deviations of all Sentinel-2 optical bands inside each forest segment. The set of computed values in all optical bands creates a habitat spectral characteristic that could be used for its identification. The Sentinel-2 satellite records 17 spectral bands from which 14 were used in our analyses for vegetation monitoring (AOT-Aerosol Optical Thickness, B01-Aerosol detection, B02-Blue, B03-Green, B04-Red, B08-Near infrared, B09-Water vapour, B11 and B12-Snow/ice/cloud discrimination, and WVP-Scene-average Water Vapour map and Vegetation classification band values B05, B06, B07 and B8A). The CLD-cloud map, SCL-Scene classification and SNW-Snow map were omitted. Each habitat is thus characterized by 56 spectral values for each date. The spatial resolution of Sentinel-2 images is up to 10 m (spatial resolution of bands B02, B03, B04, and B08 is 10 m, of bands B05, B06, B07, B8a, B11, B12, AOT, and WVP it is 20 m and of bands B01, B09 and B10 it is 60 m).

When addressing forest habitats, it sometimes occurs that native forests—priority Natura 2000 habitats and planted forests (e.g., spruce in habitats 9410 and planted spruce forests)—are formed by the same tree species; thus, the values of optical bands are similar. In planted forests, in contrast with native forests, there are trees of the same age that are often planted in rows. Therefore, in addition to optical data, characteristics expressing information about the structure can be used to recognize them—we used Relative High Laplacian (RHL) values^31^. This RLH classifier was developed and implemented in NaturaSat software to detect structural differences and capture the heterogeneity (diversity) of data. The RHL value is given by the finite difference numerical approximation of the Laplace operator applied to the intensity of the smoothed image in each pixel and then by calculating the ratio of high Laplacian pixels and all pixels within the segmented region. The high Laplacian pixels are defined relative to the maximum MAX of the Laplacian in the segmented region. We denoted by RHL N% the results of the RHL classifier for the segmented region when the pixel was considered to have the high Laplacian if its Laplacian was greater than N% of MAX. Because the high Laplace operator is achieved at the points with the highest curvature of the graph of image intensity, the RHL represents the diversity of the forest observed in the optical bands.

In addition to testing the distinguishability of all four habitat groups, we were interested in which period of the year differences in spectral characteristics of habitats were the most significant. The characteristics were computed for datasets recorded in different parts of the year: spring—May 13, 2018 (acquisition time 9:50:30), and April 20, 2019 (9:40:39); summer—July 4, 2019 (9:40:41), August 31, 2019 (9:50:39), August 30, 2020 (9:50:31), and September 9, 2021 (9:50:29); autumn—October 15, 2018 (9:50:29), November 14, 2018 (9:52:49), October 15, 2019 (9:50:31), and October 17, 2019 (9:40:29).

### Data analyses

#### Cluster analyses and PERMANOVA test

To divide the relevés into habitat types, we used the cluster analysis (divisive clustering methods) computed by the TWINSPAN program tools^47^ implemented in JUICE 7.1 software^43^ with three pseudo species cut levels (at cover values 0, 5 and 25). The relevance of division was tested using PERMANOVA to confirm the significant differences in the species composition of the identified habitats.

The spectral values were processed and prepared for testing by PERMANOVAs implemented in R software^48^ (vegan package) with Euclidean distance and Bray–Curtis indices and 999,999 permutations. All four forest habitats classified by Twinspan were first analysed together. By comparing all optical bands, we also analysed when was the best season to distinguish the planted spruce forest from habitat 9410 (native spruce forest). In addition, we analysed which band values and combinations were most appropriate and which classification results were most significant. Similarly, we examined the distinguishability of the two mixed habitats, i.e., 9130 *Asperulo-Fagetum* beech forests and 9180 *Tilio-Acerion* forests of slopes, screes, and ravines.

The RHL values were prepared for testing by ANOVAs and compared by a t test implemented in R software^48^ and visualized using boxplot graphs.

#### Hausdorff distance

The results of semiautomatic and fully automatic segmentations were compared visually and quantitatively with the GPS tracks obtained in the field. For the quantitative comparison of two polygons represented by closed planar curves, the classical (maximal) Hausdorff distance^49^ and the mean Hausdorff distance were used. Both are general tools for computing the distance of curves, surfaces, and even more complicated geometrical continuous or discrete objects^32,33^. These distances were computed using the NaturaSat software tools for sets of semiautomatic segmentations versus GPS tracks and automatic segmentations versus GPS tracks.

**Figure 2:**
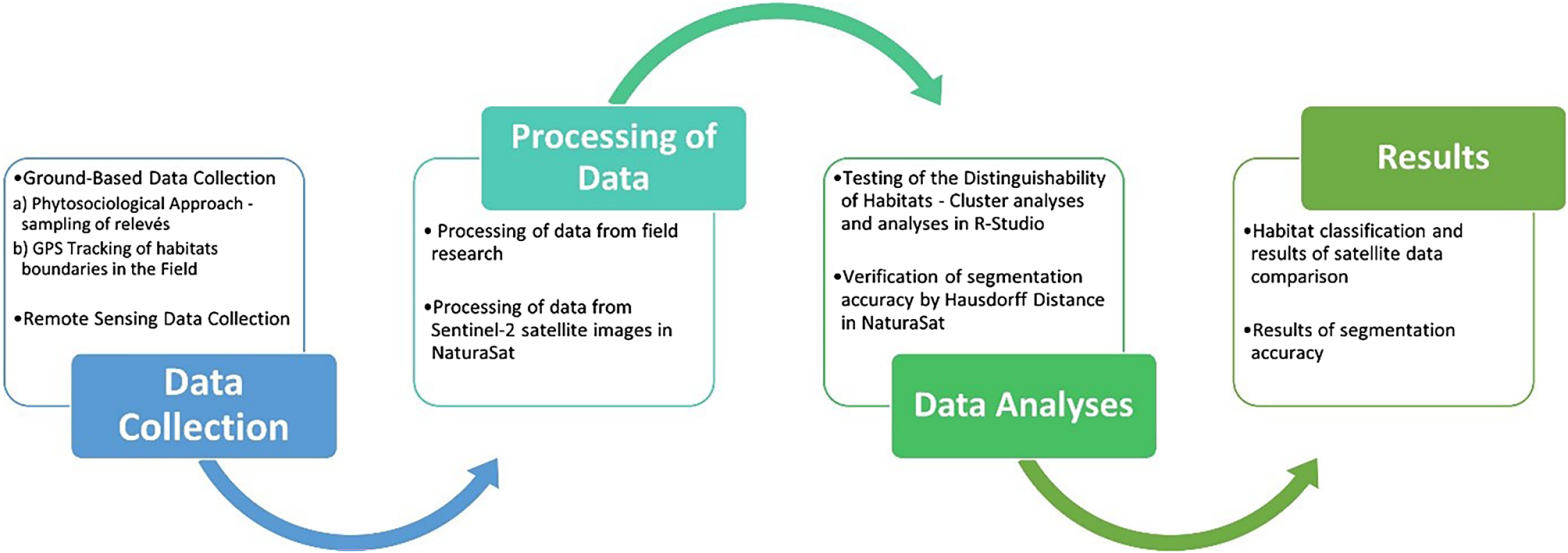
Flow chart of our research solution process.

The process of our research approach is shown in the Fig. 2.

## Results

### Habitat classification

Four different forest vegetation types (habitats) were distinguished in the case study area. From these, three of them represent Natura 2000 habitats (9130, 9180, and 9410), and one denotes planted spruce forests (secondary spruce forests) (Table 1).

**Table 1:** List of identified habitats according to the classification of the Natura 2000 system and plots that represent them in our analyses. Relevés were proportionally recorded based on the area of each habitat type in the locality.

Habitat no.	Habitat	Dominant tree species	Number of relevés/plots
9130	*Asperulo-Fagetum* beech forests	*Fagus sylvatica, Acer pseudoplatanus, Abies alba*	13
9180	*Tilio-Acerion* forests of slopes, screes, and ravines	*Fagus sylvatica, Acer pseudoplatanus, Acer platanoides, Fraxinus excelsior, Ulmus glabra*	29
9410	Acidophilous *Picea* forests of the montane to alpine levels (*Vaccinio-Piceetea*) (spruce forests)	*Picea abies, Sorbus aucuparia*	7
–	Planted spruce forest (secondary spruce forests)	*Picea abies*	16

**Figure 3:**
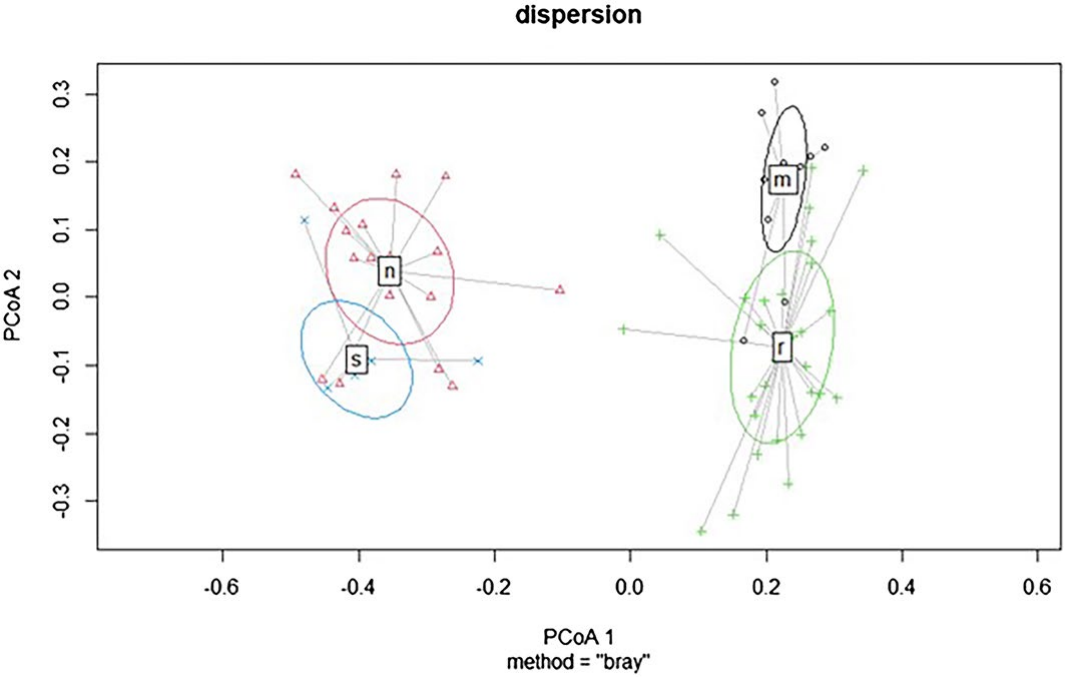
A significant differentiation of grouped relevés based on species composition showing relationships between target habitats. Abbreviations: s – 9410 – Acidophilous *Picea* forests of the montane to alpine levels*,* n – planted spruce forest, m – 9130 – *Asperulo-Fagetum* beech forests, and r – 9180 – *Tilio-Acerion* forests of slopes, screes, and ravines.

**Figure 4:**
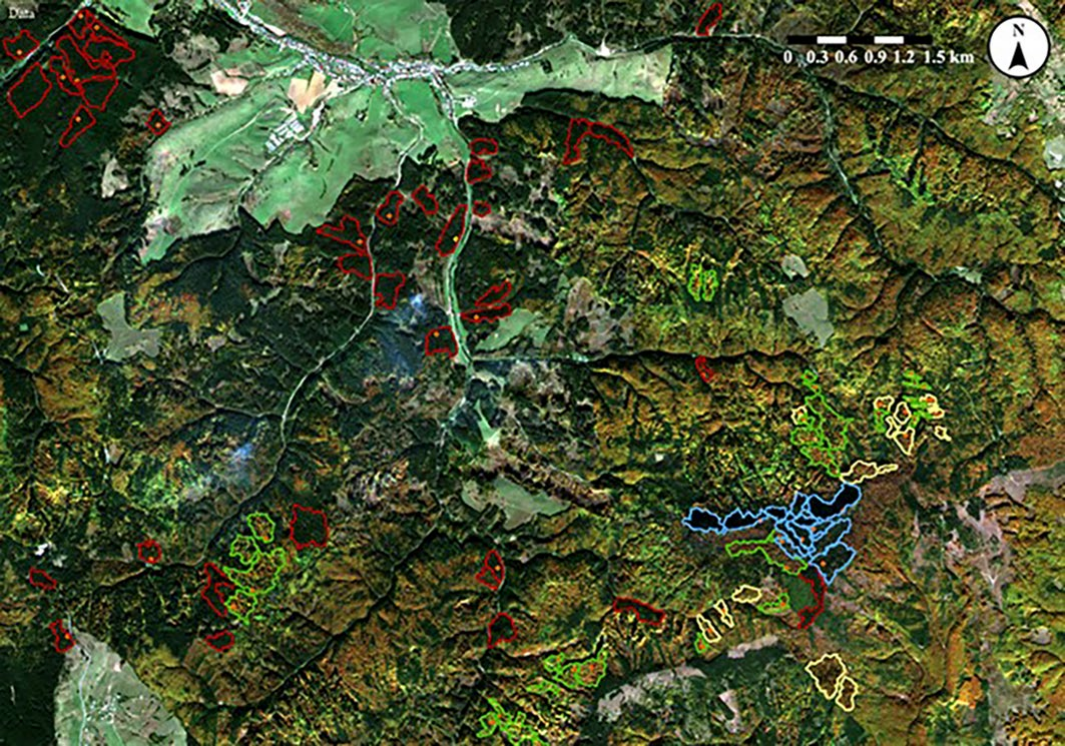
Part of the case study area in Čierny Balog with segments of habitats 9410 – Acidophilous *Picea* forests of the montane to alpine levels (blue); planted spruce forest (red); 9130 – *Asperulo-Fagetum* beech forests (yellow); and 9180 – *Tilio-Acerion* forests of slopes, screes, and ravines (green). Red dots are localities where relevés were sampled (created in software NaturaSat v1.2 ^31^, dataset from October 17, 2019; acquisition time 9:40:29, spatial resolution of Sentinel-2 satellite image is 10 m, 1 pixel on the satellite image represents 10 × 10 m on the Earth surface).

Cluster analyses showed close species relationships between coniferous and mixed forests that were divided in the first step based on the highest level of dissimilarity. Further division resulted in the creation of four clusters representing other types. The relevancy of division was confirmed by PERMANOVAs (Fig. 3). Habitats were classified according to the classification of the Natura 2000 system in Slovakia (cf ^19^.). A planted spruce forest is a nonnative habitat (although the same spruce species was planted as in natural spruce forests), and therefore, we did not code it. We used this distribution of relevés as a basis for further analyses. Part of the case study area with segments of target forest habitats is presented in Fig. 4.

The tree layer of mixed forests (9130, 9180) is dominated by *Fagus sylvatica, Acer pseudoplatanus, Acer platanoides, Fraxinus excelsior*, and *Abies alba*. Coniferous forests are dominated by *Picea abies* in 9410 with an admixture of *Sorbus aucuparia*. Mixed forests on steeper slopes and screes (9180) are species rich. They are floristically well differentiated from the other forests by the tree (sub)dominant species *Acer platanoides*, *Fraxinus excelsior*, and *Ulmus glabra* and numerous herbs of the order *Fagetalia*, such as *Actaea spicata*, *Dentaria bulbifera*, *D. enneaphyllos*, *Galium odoratum, Mercurialis perennis,* and *Pulmonaria officinalis,* together with the nitrophilous and nutrient-demanding species *Aegopodium podagraria, Geranium robertianum*, *Impatiens noli-tangere*, *Lunaria rediviva*, *Stachys sylvatica*, and *Urtica dioica*. In mixed *Fagus sylvatica-Abies alba* forests (9130), all these species are absent (they are negatively differentiated). A higher frequency and dominance of *Abies alba* is a typical feature of these forests. Spruce forests differ from mixed ones by several acidophilous species: * Avenella flexuosa*, *Hieracium murorum*, and *Vaccinium myrtillus*. Native spruce forests (9410) are typical of numerous mountain to (sub)alpine plants (e.g., *Calamagrostis villosa, Homogyne alpina*), some of which are diagnostic species of the tall herb vegetation of nutrient rich and moistened habitats of the class *Mulgedio-Aconitetea* (*Adenostyles alliariae, Cicerbita alpina, Dryopteris dilatata, Gentiana asclepiadea, Luzula sylvatica,* and *Ranunculus platanifolius*). In the spruce plantations, most of these species are absent. On the other hand, the stands are enriched by *Asarum europaeum, Dentaria bulbifera, Mercurialis perennis,* and *Viola reichenbachiana*—typical species of the order Fagetalia surviving from previous native broad-leaved or mixed forests. Forest clearing species of the class *Epilobietea angustifolii*, such as *Corylus avellana, Digitalis grandiflora, Fragaria vesca, Hypericum maculatum*, and *Rubus hirtu*s*,* represent succession residuals after cutting original forests*. Picea abies* frequently dominates in the herb layer.

Within the NaturaSat software environment, we were able to identify 107 segmented areas using the Sentinel-2 satellite images according to the procedure described in the methodology section (Fig. 3).

**Figure 5:**
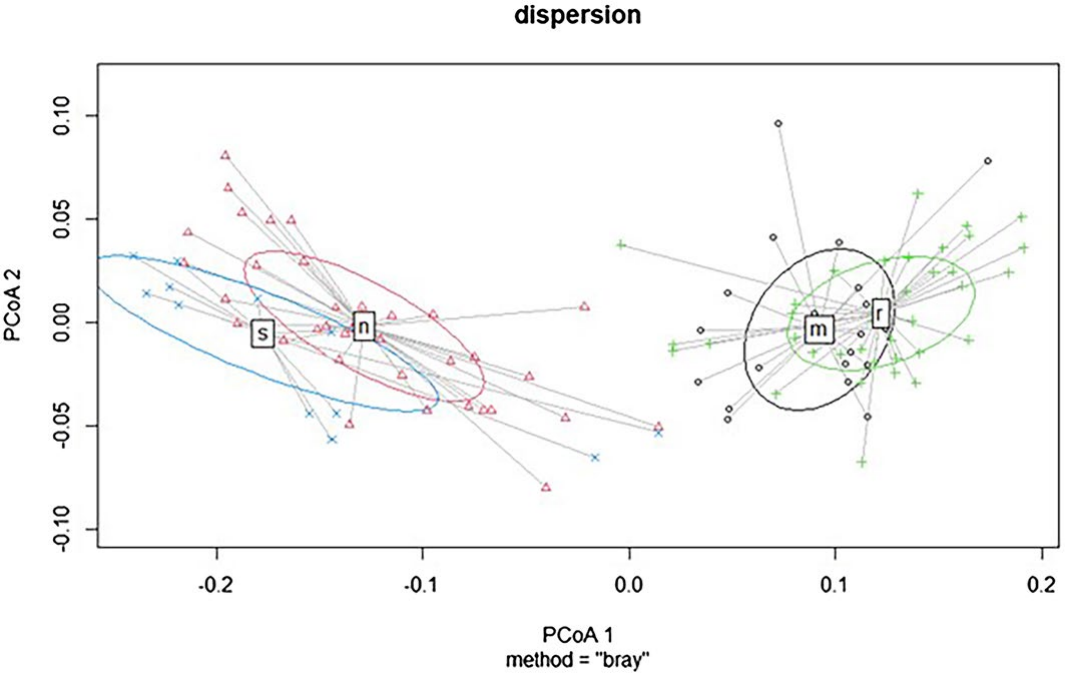
A significant differentiation of target forest types based on various combinations of optical bands extracted from Sentinel-2 data (obtained on May 13, 2018), where the P value is less than 0.001. Abbreviations: s – 9410 – Acidophilous *Picea* forests of the montane to alpine levels*,* n – planted spruce forest, m – 9130 – *Asperulo-Fagetum* beech forests, and r – 9180 – *Tilio-Acerion* forests of slopes, screes, and ravines.

By analysing the target habitats, all selected optical values described in the methods were used. The results of additional PERMANOVAs (the P values were less than 0.001, unless otherwise stated) confirmed the assumption that target forest habitats can be recognized remotely (Fig. 5).

The distinguishability of coniferous habitats and those in which deciduous trees were more numerous was confirmed at a high significance level. Consequently, these habitats were analysed separately to provide better insight into specific differences.

**Figure 6:**
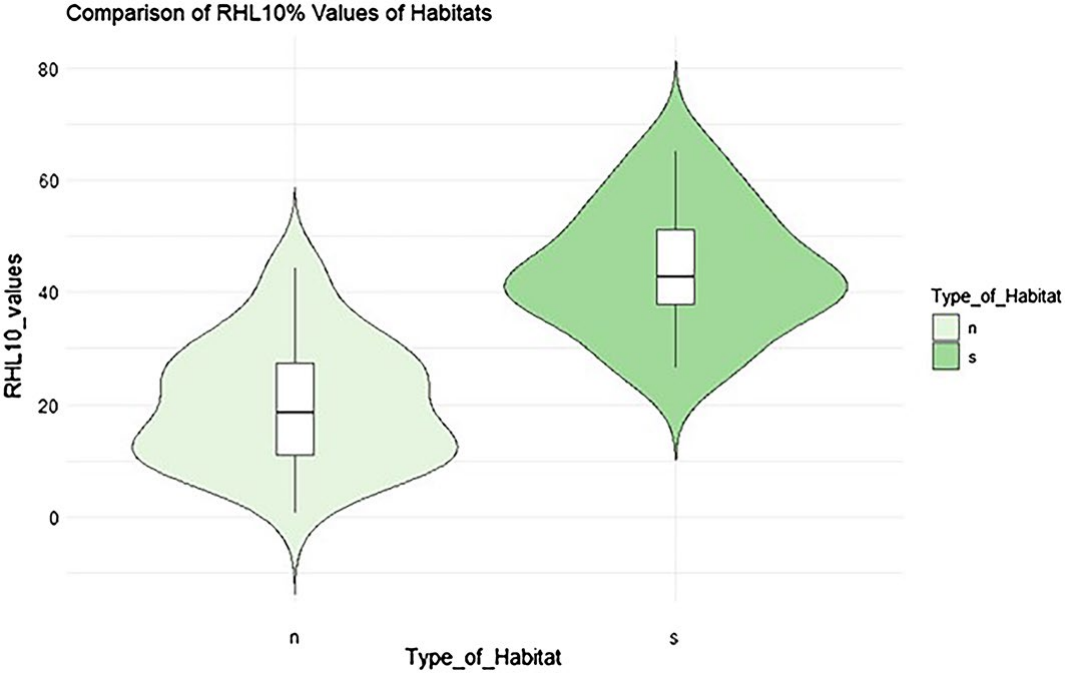
Differentiation of RHL 10% of selected forest types in the B04-Red optical band intensity value in August (dataset from August 31, 2019). On the vertical axis are values of RHL. The P value is less than 0.001. Abbreviations: n – planted spruce forest and s – 9410 – Acidophilous *Picea* forests of the montane to alpine levels.

To test the usage of RHL values to distinguish between 9410 spruce forest and planted spruce forest (forest areas older than 80 years), RHL values were calculated for 48 segments of the tested habitats. Segments of spruce forests (s) always had higher RHL values, while segments of planted spruce forests (n) had lower and more variable values of RHL (Fig. 6).

**Figure 7:**
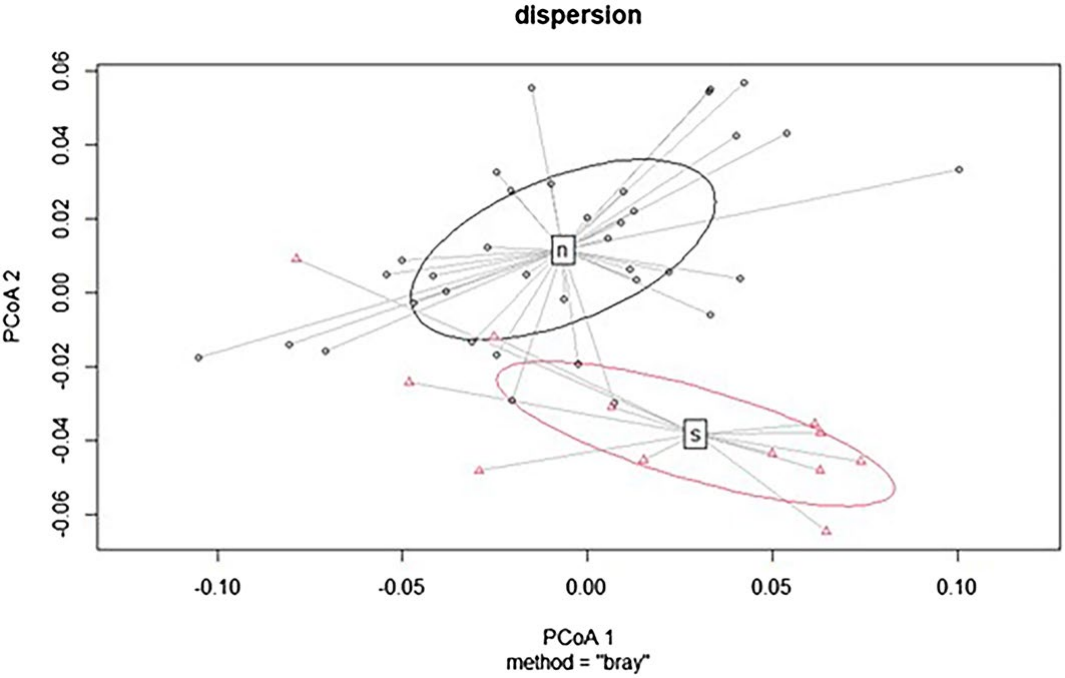
Differentiation of two coniferous forest types using mean, max, min, and Std RHL-10% (data from April 20, 2019), where the P value is less than 0.001. Abbreviations: s – 9410 – Acidophilous *Picea* forests of the montane to alpine levels and n – planted spruce forest.

In the analyses of coniferous forests, we also added the values of the RHL 10% parameter to the values of mean, max, min, and Std, as it turned out that the results of this combination of analysed values were the most significant. The period of early spring (end of April, the beginning of May) and late autumn (November) seemed to be most suitable (Fig. 7).

**Figure 8:**
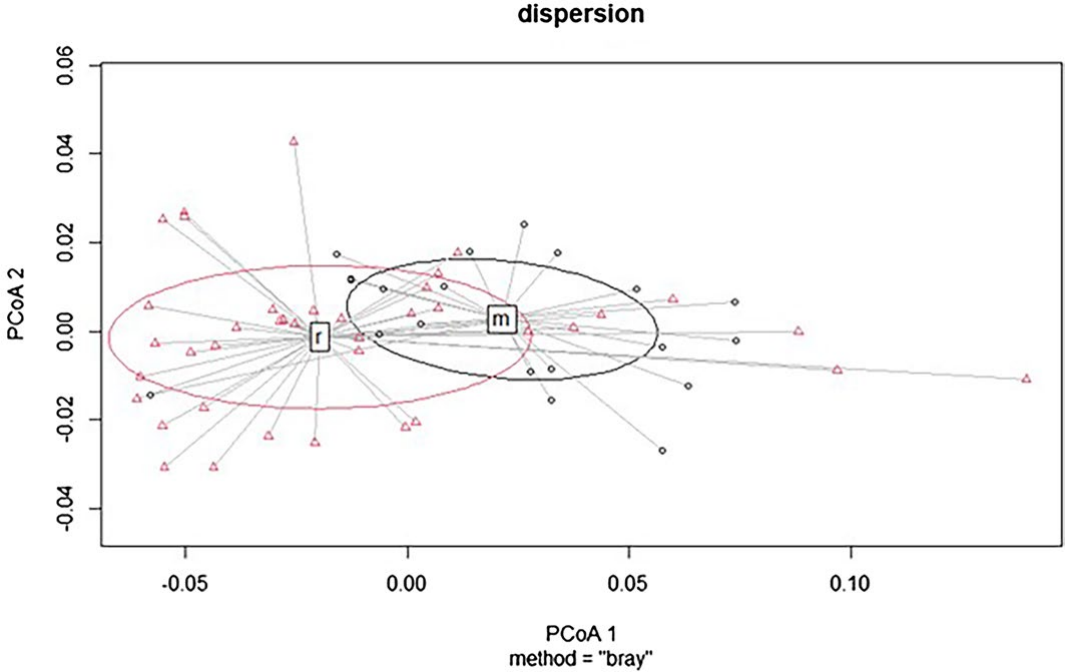
Differentiation of two mixed habitats using mean and max optical values (data from August 31, 2019), where the P value is less than 0.01. Abbreviations: m – 9130 – *Asperulo-Fagetum* beech forests and r – 9180 – *Tilio-Acerion* forests of slopes, screes, and ravines.

The most significant combination of mean and max values distinguished deciduous habitats. The summer aspect (end of August) seemed to be the most suitable for this purpose (Fig. 8).

### Segmentation accuracy

#### Accuracy of semiautomatic segmentation methods

The accuracy of semiautomatic segmentation, i.e., comparison of semiautomatic segmentation and GPS tracks by means of the Hausdorff distance, is presented in Table 2.

**Table 2:** Results of 24 semiautomatic segmentation and belonging GPS tracks compared by the Hausdorff distance (values given in meters). Abbreviations to habitats: n – planted spruce forest, m – 9130 *– Asperulo-Fagetum* beech forests, and r – 9180 – *Tilio-Acerion* forests of slopes, screes, and ravines.

Habitat	Relevé No.	Mean Hausdorff distance [m]	Maximal Hausdorff distance [m]
r	769212	7.20	22.77
r	769214	8.47	29.92
r	769220	4.91	17.67
r	769229	13.53	37.15
r	769235	11.33	36.67
r	769244	9.69	26.84
r	769245	6.98	20.46
r	769259	11.31	48.63
Habitat average		9.18	30.02
m	769209	6.92	17.48
m	769213	12.74	49.37
m	769215	5.04	24.01
m	769216	7.12	20.22
m	769222	8.56	26.32
m	769234	6.75	26.73
m	769236	7.32	19.41
m	769266	6.18	20.08
Habitat average		7.58	25.45
n	769205	11.27	31.74
n	769206	8.13	24.39
n	769207	10.39	35.15
n	769208	9.48	27.10
n	769228	12.06	42.18
n	769233	8.24	34.16
n	769254	8.09	22.02
n	769258	10.97	27.61
Habitat average		9.83	30.54
Average overall		8.86	28.67

The mean Hausdorff distance was on average 8.86 m, which is smaller than the spatial resolution (10 m) of Sentinel-2 data. This indicates that by using semiautomatic segmentation, we were able to detect habitat borders more accurately as the image resolution allowed this. The maximal Hausdorff distance was on average approximately 28.67 m (less than 3 pixels).

#### Accuracy of automatic segmentation methods

In the next part of our research, we focused on the automatic segmentation of selected habitats. The results of automatic segmentation and GPS tracks compared by the Hausdorff distance are presented in Table 3.

**Table 3:** Results of automatic segmentation and GPS tracks compared by the Hausdorff distance (values given in meters). Abbreviations to habitats: n – planted spruce forest, m – 9130 *– Asperulo-Fagetum* beech forests, and r – 9180 – *Tilio-Acerion* forests of slopes, screes, and ravines.

Habitat	Relevé No.	Mean Hausdorff distance [m]	Maximal Hausdorff distance [m]
r	769212	11.30	60.05
r	769214	9.30	29.58
r	769220	9.24	37.54
r	769229	14.41	34.13
r	769235	8.81	30.96
r	769244	17.45	77.86
r	769245	17.42	73.02
r	769259	14.86	56.34
Habitat average		12.85	49.93
m	769209	14.04	56.48
m	769213	18.68	56.54
m	769215	6.71	18.45
m	769216	8.05	35.54
m	769222	18.12	46.13
m	769234	19.14	85.89
m	769236	12.73	43.09
m	769266	9.35	32.43
Habitat average		13.35	46.82
n	769205	11.91	37.97
n	769206	12.87	33.76
n	769207	11.32	45.92
n	769208	15.36	50.45
n	769228	22.51	64.99
n	769233	22.08	64.69
n	769254	19.68	90.58
n	769258	25.75	116.82
Habitat average		17.68	63.15
Average overall		14.63	53.30

The average mean Hausdorff was 14.63 m, which was only slightly more than the spatial resolution of the satellite data. The maximal Hausdorff distance was, on average, approximately 53.3 m, which represents 5 pixels. The highest differences could be found in the areas with ecotone zones, where forest patches were connected to surroundings by shrub zones or in segments that were connected to a similar type of vegetation, separated in the field only by a natural barrier (narrow forest road, boulders, or change of slope orientation), which occurs in this type of habitat and is typical for this area but faintly recognizable on satellite images with a resolution of 10 m.

**Figure 9:**
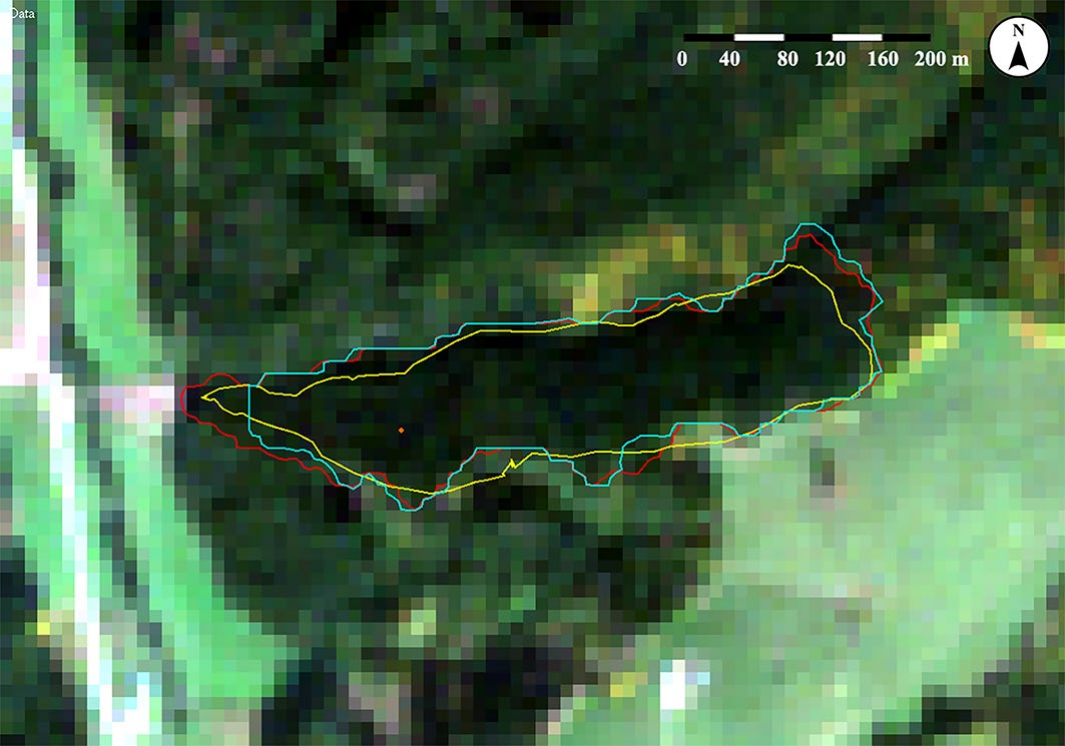
Visual comparison of three curves, GPS track (yellow), semiautomatic segmentation (red), and automatic segmentation (turquoise), for the segment of planted spruce forest, relevé 769,205 (created in software NaturaSat v1.2 ^31^, October 17, 2019; acquisition time 9:40:29, spatial resolution of Sentinel-2 satellite image is 10 m, 1 pixel on the satellite image represents 10 × 10 m on the Earth surface).

An example of a visual comparison of semiautomatic, automatic, and GPS curves is presented for the segment of habitat 9180 *Tilio-Acerion* forests of slopes, screes, and ravines, relevé IJ4299 (Fig. 9). The figure was obtained using NaturaSat software (a dataset from October 17, 2019).

The mean Hausdorff distances of segment of planted spruce forest relevé 769,205 were 11.3 m (comparison of semiautomatic segmentation and GPS track), and 11.9 m (comparison of automatic segmentation and GPS track), and 4.8 m (result of comparison between automatic and semiautomatic segmentation), which is very close and even less than the pixel resolution of the Sentinel-2 data.

## Discussion

During our case study, it was confirmed that remote sensing (RS) can make botanical and ecological research and mapping more efficient. To achieve our aims, long-term development of technology (satellites) and image processing (software) was needed. RS technology has been popular since the 1970s because of its incredible opportunities, low acquisition costs, and high-utility data collection. After considerably advanced interpretation of geography, geology, and agriculture, the observation of land cover was one of the priorities, especially after the first satellite from the LANDSAT family, ERTS-1 (Earth Resources Technology Satellite; it was later renamed LANDSAT-1), was launched in 1972^50^. Rapidly developing RS techniques were adopted to monitor land surface types such as agricultural land, water bodies, abandoned land, forests, and built areas. Launching the European Sentinel-2 satellites opened a new era in the application of freely available remotely sensed data in forestry^13^. With improved resolution, availability of multispectral data, and advanced data-processing techniques, RS offers more profound insight into land cover categories—different crop fields^51^, meadows with various management styles^52^, and different forest types (e.g.,^12,53^) have been identified successfully. RS has been utilized in agriculture (e.g., detection and mapping of weeds in agricultural crops^54–56^), forest management (e.g., RS of forest insect disturbances^57^), and assessing sustainable development^58^. Using RS for nature conservation is still a major challenge due to the complexity of nature. Ecosystems representing Natura 2000 habitats are complex plant communities, including tree, shrub, and herb layers, together with typical fauna. Mapping specific ecosystems, such as Natura 2000 habitats, has been demanding for remote sensing techniques in the past and have fallen short^59^. The monitoring of habitats is a significant part of climate change monitoring and research challenges^60^; therefore, the present study makes an important contribution to the application of RS in nature conservation.

### Evolving curve approach versus other approaches

Many studies have recently focused on recognizing different habitats using various spatial scales and pixel-based approaches with hard or fuzzy classification (e.g.,^31,61^). For such research, obtaining a high-quality training dataset is necessary. When mapping more pronounced units, basic field observations could be used; however, Natura 2000 habitats can be precisely identified mostly by the sampling of phytosociological relevés containing information about plant species composition and structure (cf ^40,41^.). In our study, the pixel-based approach was replaced by a new, evolving curve approach that allows obtaining the exact area by automatic segmentation with phytosociological relevés as a starting point. This unique interconnection between traditional ground-based information and remote sensing gives us the advantage of exact affiliation of the segmented area into Natura 2000 habitat, confirmed by species composition. The approach was tested in a case study area in Čierny Balog, but the same methods could be used in different parts of Slovakia or Europe where phytosociological relevés of studied habitats occur, which suggests strong potential for the use of this method.

### Resolution

It is well known that the spatial resolution of imagery must be carefully chosen when the spatial scale of the pattern under consideration is concerned^62^. Remote sensing data with higher resolution, such as ALS or UAV imagery, have considerable potential (e.g.,^63^). However, their disadvantages may be their higher costs and irregular, one-time only or long time between repeated data acquisitions^8^ and limitations to the target areas and vegetation types. On the other hand, many recent studies focusing on satellite data (e.g.,^64^) addressed very distinct units and pronounced gradients. At the same time, our results show that freely available multispectral remote sensing data can be used to identify some types of forests at the scale of single Natura 2000 habitats that until now were not determined based on satellite images. The results of automatic segmentation in our dataset have an accuracy of approximately 15 m and the results of the semiautomatic segmentation have an accuracy of only approximately 9 m, which is less than the pixel resolution of Sentinel-2 data (1 px = 10 m).

Mapping Natura 2000 forest habitats is a major challenge. Biotopes belonging to the Natura 2000 network are often more complex, have a transitional ecotone area with smaller trees and shrubs, or are adjacent to forests with a similar type of vegetation and are separated by obstacles visible only in the terrain (e.g., rocks, forest roads, or changes of slope). Based on field research, we also noticed these limitations in semiautomatic segmentation, but they may not be easily recognizable on satellite images and are not considered in automatic segmentation. There is also a difference between the tree trunk and tree crown/branches. The tracking of boundaries in the field may be different, especially when the trees are tall and/or the trees of different habitats are touching. The combination of these segmentation methods allows the necessary simplification and acceleration, which could be part of habitat mapping and monitoring for both scientists and conservation needs.

The methods used were able to reach the accuracy of the pixel resolution of Sentinel-2 data, which are suitable for forest habitat mapping and identification; however, the methods have large potential when using data with a ALS or UAV resolution. After proper calibration, our methods have the potential to achieve pixel resolution in these cases.

### Accuracy

The overall accuracy of different methods at diverse scales of target phenomena is very variable. Simultaneously, it is complicated to compare the evolving curve approach to pixel-based approaches. Our accuracy is based on the comparison of whole areas identified by GPS tracks and segmentation curves situated in forested areas with exactly defined Natura 2000 units. Commonly used approaches focus on the selection of a network of points in the whole landscape—from afforested sites to abandoned land.

The highest accuracy values are reached in the case of broadly defined landscape units or when basic forest cover is mapped. A study from a small part of Vietnam^65^ where Sentinel-2 imagery was used and three classification methods were compared (random forest, k-nearest neighbour, and support vector machine) showed an overall accuracy ranging from 90 to 95% when six land cover classes were mapped (residential, impervious surface, agriculture, bare land, forest, and water). By using multispectral data together with the digital elevation model, identification of forest cover was performed with high accuracy (92–98%), while for forest types, accuracy was more variable (83–97% ^13^,). Forest cover and its changes were mapped in Paraguay with accuracy varying from 85% (in 2003–2004) to 93% (in 2001–2002)^66^. Within our study, the cover of a single forest habitat type was identified against other forests, and the area that NaturaSat software could find by automatic segmentation using only optical multispectral data differed from the forest patch area by an average of 15%; the minimum value was only 0.44% (i.e., accuracy up to 99%). The borders of forest differed by 1–5 pixels, where the mean Hausdorff distance was 14.6 m, which was only 1.5 pixels of Sentinel-2 resolution between GPS-tracked and automatically segmented areas. Using semiautomatic segmentation, applying data only from Sentinel-2, the area of the target types of forest habitats was identical to the area obtained by GPS, on average, up to 90%.

Natura 2000 forest habitats are defined by more detailed criteria than most forest type classifications used in the abovementioned studies. Land cover maps tend to have high accuracy (e.g., 86% ^67^,), where pastures and bodies of water tend to be correctly classified, while forest categories tend to have a high degree of inaccuracy. Novel approaches, such as the presented evolving curve method focused on forest identification by remote sensing, are essential for improving land cover maps. In tropical forests (e.g., in Ghana ^68^;), we commonly encounter an accuracy of approximately 90–93%^68^. Forest plantations have been successfully identified in numerous examples, mainly from tropical areas in India^69^, Brazil, and Senegal, with accuracies varying between 83 and 96%^70,71^.

In the case of a higher success rate, additional information, such as the digital elevation model, NDVI, or structural data, was added to the analyses, or multispectral data were used. During more complex forest mapping in China, where ten types of natural forests were distinguished by multispectral temporal data and digital elevation models, the accuracy was 82%, which is markedly lower than that in simple forest plantations. Within our approach, only optical multispectral information was applied. Using phytosociological relevés as starting points is a novel method that brings a very high success rate in identifying strictly defined forest habitats that were never identified with such accuracy before. If we would like to focus on a wider area, the Special Areas of Conservation Ďumbierske Tatras (SKUEV0302 Ďumbierske Tatry) and Kráľovohoľské Tatras (SKUEV0310 Kráľovohoľské Tatry) are located nearby, where the same forest habitats as in the territory of Čierne Balog occur, and for reasons of protection and management of these protected areas, it is necessary to ensure the monitoring of these habitats. This data will be part of a wider data set contained also further habitats forming training data set for a deep learning network that will be able to identify protected forest habitats in Slovakia^72^. Moreover, this approach is particularly applicable in European countries, e.g. the Carpathian, Alpine and Pannonian biogeographical regions with a long tradition of phytosociological approaches, although the methods could be transferred anywhere.

## Conclusion

Our study presents an example of the potential of a new approach using an evolving curve on Sentinel-2 satellite images when identifying forest habitat type borders. The irreplaceable role of ground-based data from vegetation databases (cf. Chytrý et al. 2016, sPLot^73^) was confirmed in this study. The interconnection between the phytosociological approach and remote sensing techniques brings new possibilities for the exact mapping of forest habitats and can contribute to enhancing many future studies in vegetation and landscape ecology, as well as nature conservation strategies.

## Supplementary Information

Supplementary Information 1.

Supplementary Information 2.

Supplementary Information 3.

Supplementary Information 4.

## Data Availability

The datasets used and/or analysed during the current study available from the corresponding author on reasonable request.
